# The TELE-DD project on treatment nonadherence in the population with type 2 diabetes and comorbid depression

**DOI:** 10.1038/s41598-021-87410-9

**Published:** 2021-04-26

**Authors:** Juan Francisco Roy, María Luisa Lozano del Hoyo, Fernando Urcola-Pardo, Alicia Monreal-Bartolomé, Diana Cecilia Gracia Ruiz, María Mercedes Gómez Borao, Ana Belén Artigas Alcázar, José Pedro Martínez Casbas, Alexandra Aceituno Casas, María Teresa Andaluz Funcia, Javier García-Campayo, María Teresa Fernández Rodrigo

**Affiliations:** 1 Universidad Internacional de La Rioja, 26006 Logroño, Spain; 2 Centro de Salud Las Fuentes Norte, Servicio Aragonés de Salud (SALUD), 50002 Zaragoza, Spain; 3 Department of Physiatry and Nursing, Faculty of Health Sciences, University of Zaragoza, 50009 Zaragoza, Spain; 4 Water and Environmental Health Research Group (DGA-B43-20R), Zaragoza, Spain; 5 Aragon Institute for Health Research, IIS Aragon, 50009 Zaragoza, Spain; 6 Primary Care Prevention and Health Promotion Research Network, RedIAPP, 28029 Madrid, Spain; 7 Department of Medicine and Psychiatry, Faculty of Medicine, University of Zaragoza, 50009 Zaragoza, Spain; 8 Centro de Salud Sagasta, SALUD, 50006 Zaragoza, Spain; 9 Miguel Servet University Hospital, Zaragoza, Spain; 10 Centro de Salud San Pablo, SALUD, 50003 Zaragoza, Spain

**Keywords:** Psychology, Endocrinology, Health care, Health services

## Abstract

Diabetic patients have increased depression rates, diminished quality of life, and higher death rates due to depression comorbidity or diabetes complications. Treatment adherence (TA) and the maintenance of an adequate and competent self-care are crucial factors to reach optimal glycaemic control and stable quality of life in these patients. In this report, we present the baseline population analyses in phase I of the TELE-DD project, a three-phased population-based study in 23 Health Centres from the Aragonian Health Service Sector II in Zaragoza, Spain. The objectives of the present report are: (1) to determine the point prevalence of T2D and clinical depression comorbidity and treatment nonadherence; (2) to test if HbA1c and LDL-C, as primary DM outcomes, are related to TA in this population; and (3) to test if these DM primary outcomes are associated with TA independently of shared risk factors for DM and depression, and patients’ health behaviours. A population of 7,271 patients with type-2 diabetes and comorbid clinical depression was investigated for inclusion. Individuals with confirmed diagnoses and drug treatment for both illnesses (n = 3340) were included in the current phase I. A point prevalence of 1.9% was found for the T2D-depression comorbidity. The prevalence of patients nonadherent to treatment for these diseases was 35.4%. Multivariate analyses confirmed that lower diabetes duration, increased yearly PCS visits, HbA1c and LDL-C levels were independently related to treatment nonadherence. These findings informed the development of a telephonic monitoring platform for treatment of nonadherence for people with diabetes and comorbid depression and further trial, cost-effectiveness, and prognostic studies (phases II and III).

## Introduction

Diabetes Mellitus (DM) is currently a global health threat affecting more than 380 million people, an alarming number predicted to reach 590 million in the year 2035^1^. DM is often accompanied by serious short-term consequences, such as hypoglycaemia, hyperglycaemia, and micro- and macrovascular long-term complications that substantially increase patient’s disability and mortality rates^2^, while being the cause of more renal failure, blindness, cardiovascular disease, and amputation cases than any known illnesses^3^. The prevention and treatment of these complications, together with DM chronicity, require a complex self-management throughout the patients’ life-course, but also health professionals with a strong commitment with the patients’ education and wellbeing by promoting medication adherence, a healthy diet, physical exercise, and blood glucose control, for a better prognosis^4^. If DM is undiagnosed or untreated, it will significantly increase cardiovascular risk and reduce life expectancy while increasing the risk of hospital admissions, and health costs^5^.

Daily and chronic issues lead to increased emotional distress in diabetic patients when compared to the general population, being clinical depression the most frequent comorbid disease^6–9^ and leaving no doubts about the bidirectional association between them^10,11^. Moreover, the risk of depression relapse increases progressively with each new episode, particularly and negatively impacting DM patient’s prognosis^12^. Specifically, diabetic patients are on a two-fold increased risk for a major depression disorder (MDD) in the general population^13^, a injurious comorbidity associated with adverse health issues as higher HbA1c levels and reduced optimal self-care behaviours^14,15^. In subjects with overweight or obesity, lowering body weight by 5–10% is related with bettered insulin sensitivity, improved glycaemic control, lower triglycerides, and elevated HDL-C^16^. Besides, the International Atherosclerosis Society and the European Society of Cardiology^17,18^ recommend low-LDL levels of 100 mg /dl in diabetics without cardiovascular disease (CVD). Besides, an LDL cholesterol goal lower than 70 mg/dL should be achieved in T2D patients with high cardiovascular burden or risk (CVD or associated risk factors), which has been as well related to a higher risk of new or recurrent clinical depression episodes^19^. In addition, previous studies indicated a significant correlation between hypertension and depression in old adults in community-based studies^20^ and indicated that a diastolic blood pressure difference of 5 mmHg significantly reduces the complications of death from diabetes and cerebral infarcts and the development of microvascular complications^21^. Physical inactivity, overweight/obesity, daily smoking, and hypertension have recently been demonstrated to be shared, and unique risk factors of DM and depressive disorders^22^. Diabetes and depression comorbidity significantly increase micro- and macrovascular complications^23,24^, elevated health care use^25^, and death rates^26^. In fact, individuals diagnosed with DM and comorbid depressive disorder have 2,5 higher odds to die in the next eight years than patients with only DM or depression^26^.

An adequate treatment adherence (TA) occurs when patients' behaviour matches regular prescription and advice from health professionals, as medication intake, diet self-monitoring, and/or executing lifestyle changes^27^. In patients with DM, TA involves the optimization of blood glucose levels and maintaining a healthy lifestyle and is considered a determinant factor to improve prognosis by reducing disease outcomes, as incident complications, and death^28,29^. A meta-analysis of 513 studies across 50 years indicated a mean TA for DM of 67%, being the lowest TA of the 17 main diseases analysed and estimated 7.6 million of visits to the physician probably ending in nonadherence^30^, additionally, a median prevalence of 53% was found for unipolar depression treatment non-adherence^31^. Treatment nonadherence results in catastrophic loss in human lives, and also in medical care costs, in fact, the American Diabetes Association (ADA) combined and updated the estimation for the economic burden of DM for all ages, including direct and indirect cost, reaching $404 billion in 2017^32^. Based on prior research, the objectives of the present report are: (1) to determine the point prevalence of T2D and clinical depression comorbidity and treatment nonadherence; (2) to test if HbA1c and LDL-C, as primary DM outcomes, are related to TA in this population; and (3) to test if these DM primary outcomes are associated with TA independently of shared risk factors for DM and depression, and patients’ health behaviours.

## Results

### Prevalence data for T2D and depression comorbidity and TA (objective 1)

**Figure 1:**
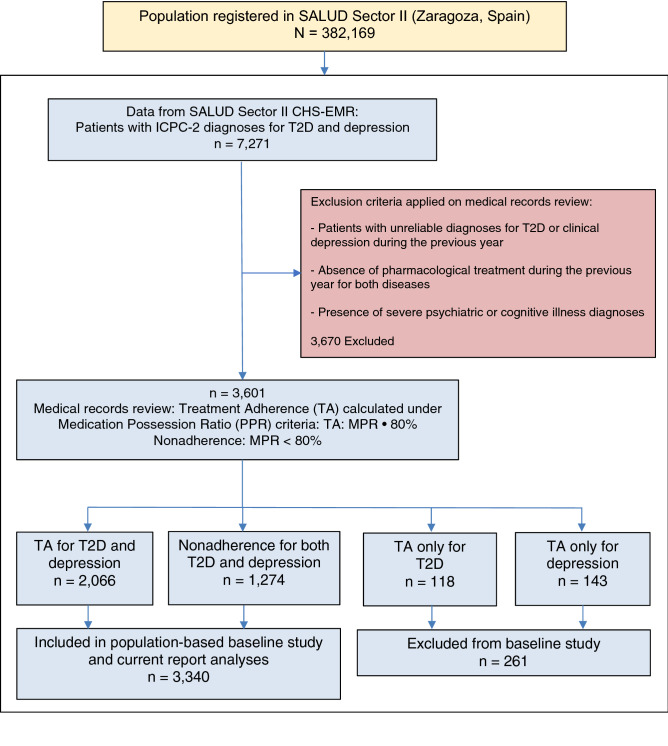
TELE-DD project flowchart.

The population of Zaragoza (Spain) from the Aragonese Health Service (SALUD) Sector II included 382,169 people of which 7271 (Fig. 1) were identified from the Community Health System-Electronic Medical Records (CHS-EMR) as patients diagnosed with T2D and concurrent clinical depression, resulting in a comorbidity crude point prevalence of 1.9%. After reviewing the medical records of these patients, those who had unreliable diagnoses for T2D or clinical depression or did not receive pharmacological treatment during the previous year for both diseases, or suffered severe psychiatric or cognitive illness, were excluded from the study (n = 3670), while halving the comorbidity point prevalence to 0.94%. To obtain the point prevalence of nonadherence *vs* TA, the remaining 3601 patients were grouped with regard to TA criteria for T2D, clinical depression, or both, by using the Medication Possession Ratio or MPR, defining TA as MPR ≥ 80% and nonadherence as MPR < 80%. One hundred and eighteen patients adherent only to pharmacological treatment of diabetes and 143 to pharmacological treatment of clinical depression were additionally excluded to avoid bias when analyzing the association between quality of adherence and study primary outcomes and covariates. Finally, 2066 patients (point prevalence = 57.4%) that showed TA and those 1274 patients that presented nonadherence or non-TA (point prevalence = 35.4%) to both pharmacological treatments were included in the final TELE-DD phase I baseline study (n = 3340) and current report analyses.

### Patients characteristics

The mean (Standard Deviation, SD) age of the baseline study patients (n = 3340) with T2D and comorbid depression, with or without TA for both antidiabetics and antidepressants pharmacological treatment, was 72.37 (11.95) years (Table 1). From those 3340 patients 69% were women with 90.2% of non-smokers, there was an average of 11.9 (8.6). Primary Care Specialist (PCS) consultation visits per year, and those for nurse consultation supposed a mean of 9.0 (11.5) visits during the year preceding the baseline assessment. Patients consumed an average of 6.4 (2.9) drugs.

**Table 1:** Baseline TELE-DD population characteristics of patients with type-2 diabetes and comorbid clinical depression by treatment adherence.

Variables^†^	TA	Non-TA	p (95%)
Age, years (Mean, SD)	72.57 (11.80)	72.04 (12.19)	.210
**Sex (n, %)**			
Female	1446 (70.0%)	859 (67.4%)	.119
Male	620 (30.0%)	415 (32.6%)	
Diabetes duration, years (Mean, SD)	8.80 (5,63)	8.38 (5.62)	.034
Depression duration, years (Mean, SD)	8.31 (5.20)	7.96 (4.99)	.058
Nurse appointments, yearly Mean, SD)	8.98 (11.11)	9.00 (12.18)	.959
PCS appointments, yearly (Mean, SD)	11.66 (8.39)	12.14 (8.76)	.116
Drug intake (No.) (Mean, SD)	6.34 (2.88)	6.36 (2.96)	.893
**Smoking status, current**			
No	1873 (90.7)	1.141 (89.6)	.299
Yes	193 (9.3)	133 (10.4)	
BMI (Mean, SD)	30.70 (5.45)	30.38 (5.28)	.094
**BMI, WHO Criteria (n, %)**			
Normal	259 (12.5)	170 (13.3)	.796
Pre-obesity	781 (37.8)	486 (38.1)	
Obesity class I	607 (29.4)	375 (29.4)	
Obesity class II	289 (14.0)	175 (13.7)	
Obesity class III	130 (6.3)	68 (5.3)	
Systolic blood pressure (Mean, SD)	134.95 (16,68)	134.76 (16.99)	.756
Diastolic blood pressure (Mean, SD)	76.11 (10.05)	75.83 (10.02)	.436
HbA1c (Mean, SD)	6.99 (1.10)	7.51 (1.41)	.000
**HbA1c levels (n, %, ADA 2018 criteria)**			
< 5.7	131 (6.3)	37 (2.9)	.000
5.7–5.99	130 (6.3)	71 (5.6)	
6–6.49	474 (22.9)	238 (18.7)	
6.5–6.99	445 (21.5)	195 (15.3)	
7–7.99	506 (24.5)	305 (23.9)	
8–8.99	247 (12)	215 (16.9)	
9–9.99	104 (5)	139 (10.9)	
> 10	29 (1.4)	74 (5.8)	
LDL-C (Mean, SD)	105.97 (33.27)	109.09 (33.54)	.009
**LDL-C levels (n, %, ATP III criteria)**			
< 100	989 (47.9)	559 (43.9)	.048
100–129.99	624 (30.2)	380 (29.8)	
130–159.99	315 (15.2)	240 (18.8)	
160–189.99	105 (5.1)	71 (5.6)	
> 190	33 (1.6)	24 (1.9)	

^†^Total N = 3340 for all variables.

### Association of TA with primary DM outcomes (objective 2)

Bivariate analyses by TA indicated statistically significant differences for diabetes duration in years (*p* = 0.034), mean HbA1c (*p* < 0.001), and levels ADA 2018 standards and cut-offs, *p* < 0.001), and LDL-C mean values (*p* = 0.009) and levels (ATP III criteria, *p* = 0.048). Due to the systematic registration of CHS-EMR, and research nurse’s data collection and investigation in health centres data, there were no missing values in any variable used in the baseline population analyses.

### Hierarchical multivariate analysis of the association of primary DM outcomes with TA (objective 3)

In Table 2 a multivariate approach was used where all study variables were continuous but sex and smoking status. Unadjusted LR results confirmed significant findings from prior bivariate analyses displayed in Table 1. After further adjustment for shared T2D-depression risk factors and patients health behaviours similar findings were obtained, as shown in model 3 in Table 2. Furthermore, an inverse linear association of T2D duration in years and nonadherence was found (*p* = 0.001), showing that patients with a lesser T2D disease duration since diagnosis had an increased risk for nonadherence. On the other hand, final model 3 confirmed a direct linear relationship between the increased number of yearly PCS visits (*p* = 0.021) and in HbA1c (*p* < 0.001) and LDL-C mean values (*p* = 0.012) between patients with TA when compared with those patients with treatment nonadherence for both illnesses, indicating that increased values in those variables were significantly associated with nonadherence. Of note, Table 2 shows that T2D patients with comorbid depression had a 42% higher odds per point increase in HbA1c for nonadherence to both T2D and depression treatments when compared to those with TA. Table 3 shows the main findings of a second multivariate approach, including the categorization of primary outcomes according to well-known standards, indicating the same linear results on TA for T2D duration and physician appointments.

**Table 2:** Risk estimation for treatment nonadherence for primary DM outcomes and study covariates through hierarchical multivariate logistic regression models on diabetic patients with comorbid depression (N = 3340).

Study variables	Categories	Unadjusted			Model 1			Model 2			Model 3		
		OR	95% CI		OR	95% CI		OR	95% CI		OR	95% CI	
Age	Increased score	1.00	0.99	1.01	1.00	0.99	1.01	1.00	0.99	1.01	1.00	0.99	1.01
Sex	Female	1.00			1.00			1.00			1.00		
	Male	1.13	0.97	1.31	1.16	0.98	1.36	1.12	0.95	1.33	1.12	0.94	1.32
Diabetes duration, years	Ref	1.00						1.00			1.00		
	Increased score	0.98*	0.97	0.99				0.98**	0.97	0.99	0.98**	0.96	0.99
Depression duration, years	Ref	1.00						1.00			1.00		
	Increased score	0.98	0.97	1.01				0.99	0.98	1.01	0.99	0.98	1.01
Nurse visits	Ref	1.00						1.00			1.00		
	Increased score	1.00	0.99	1.01				1.00	0.99	1.01	1.00	0.99	1.00
PCS visits	Ref	1.00						1.00			1.00		
	Increased score	1.01	1.00	1.02				1.01*	1.00	1.02	1.01*	1.00	1.02
Drug intake (#)	Ref	1.00									1.00		
	Increased score	1.00	0.98	1.03							0.99	0.96	1.02
Smoking Status	Ref	1.00						1.00			1.00		
	Current smoker	1.13	0.90	1.43				1.16	0.90	1.49	1.15	0.90	1.48
BMI	Ref	1.00									1.00		
	Increased score	0.99	0.98	1.01							0.99	0.97	1.00
Systolic Blood pressure	Ref	1.00									1.00		
	Increased score	1.00	0.99	1.01							1.00	0.99	1.01
Blood Diastolic pressure	Ref	1.00									1.00		
	Increased score	1.00	0.99	1.01							1.00	0.98	1.01
HbA1c	Ref	1.00			1.00			1.00			1.00		
	Increased score	1.39**	1.31	1.48	1.39**	1.32	1.48	1.42**	1.34	1.50	1.42**	1.34	1.51
LDL Cholesterol	Ref	1.00			1.00			1.00			1.00		
	Increased score	1.01**	1.01	1.01	1.01**	1.01	1.01	1.01**	1.01	1.01	1.01*	1.01	1.01

Model 1 = Adjusted by age, gender, and primary DM outcomes (HbA1c and LDL Cholesterol).

Model 2 = Model 1 + T2D duration (years), depression duration (years), nurse and PCS visits, and smoking status.

Model 3 = Model 2 + # drug intake, BMI, systolic blood pressure, diastolic blood pressure.

*p < .05; **p < .01.

**Table 3:** Risk estimation for treatment nonadherence of primary DM outcomes by cut-off standards through hierarchical multivariate logistic regression models on diabetic patients with comorbid depression (N = 3340).

Study variables	Categories	Unadjusted			Model 1			Model 2			Model 3		
		OR	95% CI		OR	95% CI		OR	95% CI		OR	95% CI	
Diabetes duration, years	Ref	1.00						1.00			1.00		
	Increased score	0.98*	0.97	0.99				0.98**	0.97	0.99	0.98**	0.97	0.99
PCS visits	Ref	1.00						1.00			1.00		
	Increased score	1.01	1.00	1.02				1.01*	1.00	1.02	1.01*	1.00	1.02
HbA1c, ADA 2018 standards and cut-offs for diagnosis and outcomes	< 5.7 (Ref., Normal)	1.00			1.00			1.00			1.00		
	5.7–5.99 (Prediabetes)	1.93**	1.21	3.08	1.90**	1.19	3.03	1.89**	1.19	3.02	1.91**	1.20	3.06
	6–6.49 (Prediabetes)	1.78**	1.20	2.64	1.76**	1.18	2.62	1.75**	1.18	2.61	1.78**	1.20	2.65
	6.5–6.99 (Diabetes)	1.55*	1.04	2.32	1.54*	1.03	2.30	1.56*	1.04	2.33	1.58*	1.06	2.37
	7—7.99 (Diabetes)	2.13**	1.44	3.16	2.14**	1.44	3.16	2.22**	1.50	3.29	2.27**	1.53	3.37
	8–8.99 (Diabetes)	3.08**	2.05	4.64	3.08**	2.04	4.64	3.20**	2.12	4.83	3.27**	2.16	4.94
	9–9.99 (Diabetes)	4.73**	3.03	7.38	4.81**	3.08	7.51	5.03**	3.21	7.87	5.17**	3.30	8.11
	> 10 (Diabetes)	9.03**	5.14	15.87	8.98**	5.08	15.85	9.39**	5.31	16.62	9.60**	5.42	17.01
LDL Cholesterol, ATP III Criteria	< 100 (Ref.)	1.00			1.00			1.00			1.00		
	100–129.99	1.08	0.91	1.27	1.12	0.95	1.33	1.13	0.95	1.34	1.12	0.95	1.33
	130–159.99	1.35*	1.11	1.64	1.42**	1.16	1.74	1.40**	1.14	1.72	1.38**	1.12	1.70
	160–189.99	1.20	0.87	1.65	1.15	0.83	1.60	1.15	0.83	1.61	1.16	0.83	1.62
	> 190	1.29	0.75	2.20	1.11	0.63	1.95	1.10	0.62	1.93	1.11	0.63	1.96

Model 1 = Adjusted by age, gender, and primary DM outcomes: HbA1c, ADA criteria; and LDL Cholesterol, ATP III criteria.

Model 2 = Model 1 + T2D duration (years), depression duration (years), nurse and PCS visits, and smoking status.

Model 3 = Model 2 + : # drug intake, BMI, systolic blood pressure, diastolic blood pressure.

Only variables with significant results are displayed in this table.

*p < .05; **p < .01.

### HbA1c independent association with TA

A direct linear association was found (Table 3) for Hb1Ac when using the ADA 2018 standards and cut-offs for diagnosis and outcomes and showing that the population with prediabetes are close to reaching a two-fold increased risk of nonadherence. When patients HbA1c is situated in the first level of a T2D diagnose (6.5–6.99%; or 48–52 mmol/mol), the risk for nonadherence lowers to a 50% odds; when HbA1c increases above 7% or 53 mmol/mol that increased risk augments proportionally, from a 127% odds (OR = 2.27; CI = 1.53–3.37) on the 7–7.99% range to an 860% odds (OR = 9.6; CI = 5.42–17.01) on the group of patients with HbA1c levels above 10% (or 86 mmol/mol).

### LDL-C independent association with TA

**Figure 2:**
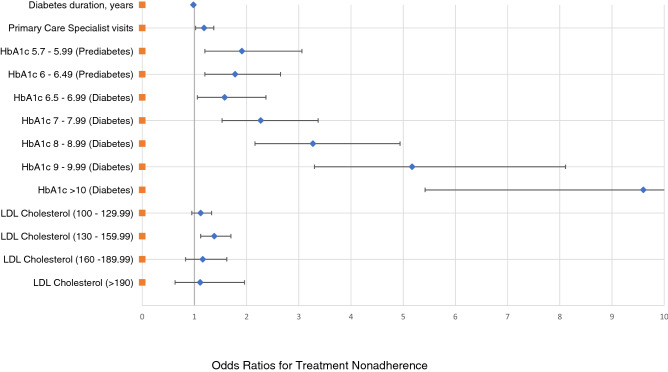
Forest plot for primary outcomes and significant covariates on treatment nonadherence in type 2 diabetes patients with comorbid depression.

Conversely, LDL-C levels according to ATP III cut-off Criteria show no significant association to TA except for the category ranging from 130 to 159.99 mg/dL, which shows a 1.4-fold increased risk of nonadherence for both T2D and depression pharmacological treatment. A forest plot in Fig. 2 displays OR and CI of the statistically significant variables of the fully adjusted model 3 shown in Table 3.

## Discussion

The baseline results show that the TELE-DD diabetic population with comorbid clinical depression is generally old and predominantly female, coincident with previous studies that reported similar characteristics and prevalence data in a similar setting^8,10^. The baseline assessment showed a 57.4% of patients with TA to T2D and depression, our data being comparable to those previously reported^33,34^. Our findings show that 35.4% of patients do not use their medication correctly, a large number considering the impact on their QOL, wellbeing and further prognosis, while coincident with DiMatteo^30^ meta-analysis finding from 513 studies across 50 years, that indicated a mean nonadherence for DM of 34%. Also, we found that lesser diabetes duration, increased primary care specialist visits, and HbA1c levels are key factors associated with nonadherence to treatment in this population with T2D and comorbid depression. In this sense, significant results have been found for the HbA1c as a continuous variable, been consistent with prior studies^35–37^. In addition, by categorizing the HbA1c values according to the ADA standards, we found that a higher risk of nonadherence is related to higher values of HbA1c. In fact, and regardless of the convenience on using continuous variables in a first multivariate approach, categorization is necessary to assess the risk of nonadherence with respect to well-known standards on HbA1c and LDL-C, and also for further comparisons with other studies while facilitating the translation of our findings to PC services. We found that patients in the prediabetes state have an increased risk of failing in both treatments between 78 and 93%, being a higher risk than those with HbA1c levels of 6.5–7%. Moreover, the risk of nonadherence has been related in our population with lesser diabetes duration and increased HbA1c values, reaching levels of concern that indicate the urgent convenience of implementing intervention and prevention programs aimed to TA increase in patients with high HbA1c levels and those with persistent or recurrent clinical depression. Findings of PCS and nurse consultation visits had similar average scores but, according to Pouwer et al.^38^, the number of consultations has a direct impact on TA and, surprisingly, our data indicate that a higher number of visits results in a worse adherence, nevertheless, patients with worse TA have worsened prognosis, as increased risk of depression relapse and DM complications, requiring further consultations than those patients with uncontrolled TA^39^, although this association may also be due to reverse causality bias. The mean number of drug intake was 6.4, similar to the data reported by Patel et al.^40^, as complexity in treatment has been reported as the main cause of nonadherence, and a consequence of the increased burden of multimorbidity, mainly due to the high number of drugs prescribed and the difficulty of handling fractionated doses^41^. Limitations: The CHS-EMR does not include data from private healthcare services, that could have been used by an estimated 16% of the study population. Specific measures for depression and DM distress are not yet systematically assessed and recorded in the CHS-EMR for DM patients, so they could not be included in current baseline analyses as covariates. Besides, our secondary data study design is cross-sectional, and all variables data (including exposures and outcomes) was obtained simultaneously, hence the direction of the association between variables cannot be accurately identified, in addition we had no access to the precise number of patients matching each exclusion criteria.

Our baseline population data show an average patient profile of an old-aged woman with nonadherence, pre-obesity or stage-I obesity, moderately high levels of LDL-Cholesterol and HbA1C levels, polypharmacy treatment, and frequent PCS visits. Bearing in mind that most of them live in poor districts with a dependency rate of 52%, and 12% of these are elders living alone, sets the urgent need of investigating and targeting this population social determinants of health^42^. In addition, the overly complex and stressful daily tasks of chronic DM self-management, and the emotional distress of elderly patients with comorbid clinical depression, also set the urgent need of health professionals personal contact, monitoring and emotional support for a higher disease self-management and TA in reducing adverse outcomes while increasing prognosis and wellbeing in diabetic patients with comorbid depression^43^. Besides, the present pandemic context due to the SARS-CoV-2 coronavirus set an immediate need for tele-health monitoring. For these reasons, the current phase I results have informed the development of a telephonic monitoring platform for treatment of nonadherence for people with diabetes and comorbid depression to be implemented in SALUD Sector II, to be tested in following phases II and III. The aim of the next study on phase II, a pragmatic RCT nested into TELE-DD population-based study, will be to test the efficacy of a proactive motivational telephonic monthly intervention, centred on a psychological and educational individualized monitoring protocol, to increase TA and disease prognosis on individuals with T2D and concurrent depression while reducing health costs, in a random stratified sample of patients from the current report phase I population. Those phase II findings, together with future 5- to 10-years follow-up prognostic study results (phase III), will allow further comparisons between RCT findings and follow-up data, to determine the potential reduction on incident complications and death rates^24^, the intervention costs-effectiveness, and will enable the TELE-DD intervention protocol to be translated to habitual primary care, specialized clinical practice, and public health prevention programs.

## Methods

### Design, setting, and baseline population

The baseline assessment of a population-based secondary data study was performed in TELE-DD Study phase I (Fig. 1), including the full population of adult (21 + years) patients diagnosed with T2D and concurrent clinical depression belonging to the 23 Health Centres from the Aragonese Health Service (SALUD) Sector II, in the city and province of Zaragoza, Aragon (Spain). These 23 Health Centres are distributed throughout the city and several neighbour districts and towns and will guarantee a both diverse and representative population of research patients. Sector II includes a total population of 382,169 inhabitants, containing most of poor (low-income) districts from the city of Zaragoza, including 33% of people and 12% of retired elders (> 65 yr) living alone and a dependency ratio of 52% relating the number of children under age 14 and elders over 65 to the working-age population.

### Patient’s eligibility and diagnostic criteria

Eligible for inclusion in the TELE-DD baseline study were all patients with concurrent T2D and clinical depression diagnoses recorded in the SALUD health electronic system to December 31st, 2015, according to the CHS-EMR registration system that uses the OMI-Primary Care (OMI-PC) computerized platform. Diagnoses correspond with the International Classification of Primary Care-2nd Edition (ICPC-2) codes T90 (type 2 diabetes) and P76 (clinical depression) and were registered and confirmed by the patient's PCS; the ICPC-2 system has the highest specificity due to its conversion structure with the International Classification of Diseases (ICD-10).

### Exclusion criteria

Unreliable ICPC-2 diagnoses for T2D or depression or absence of pharmacological treatment for both diseases during the previous year. Patients with severe psychiatric or cognitive illness diagnoses were also excluded.

### Data acquisition and management

All data for primary outcomes and covariates were obtained from the CHS-EMR, that systematically registers every patient data from all health services, including biochemical, PC, nursing, medical and other specialist services.

### Treatment adherence

Standard measurements of TA were defined through the medication possession ratio (MPR), corresponding to the number of drug units prescribed divided by the number of drug units scheduled for a specific time period^44^. This formula accounts for the rate of real-time/ scheduled time being treated: 100 × ∑ (days supplied)/365 (one year). The time in days being treated was predicted as the number of drug units prescribed during the observation year, presuming that drug dosage corresponds to one daily drug treatment during the year before the baseline assessment. When TA was calculated, a binary variable was generated as TA presence/absence under standard criteria of TA = MPR ≥ 80% and nonadherence = MPR < 80%^38,45,46^.

### Primary DM outcomes: glycosylated haemoglobin

HbA1c levels and cut points were classified according to the ADA standards of medical care on the classification and diagnosis of diabetes, as follows: Normal HbA1c < 5.7 (38 mmol/mol or lower); Prediabetes HbA1c = 5.7–6.49 (39–47 mmol/mol); and Diabetes HbA1c ≥ 6.5–6.99 (48 mmol/mol or higher)^47^.

### Low-density lipoprotein cholesterol

LDL-C values were annotated following the indications and numbers of reference from the National Cholesterol Education Program Adult Treatment Panel III^17,18^: < 100 mg/dl optimal, 100–129 mg/dl regular level, 130–159 mg/dl regular-elevated, 160–189 mg/dl elevated, > 190 mg/dl high risk.

### Covariates: Health behaviours and shared risk factors of T2D and depression

We collected data at the baseline assessment from the year of T2D diagnosis and the year of first depression diagnosis. Patient health behaviour indicators were also gathered, including the number of yearly visits to PCS and nursing professionals before the baseline assessment, current smoking status, detailed pharmacological treatment for diabetes and depression, and the number of prescribed medications. The body mass index (BMI) was also calculated as the rate of weight in kilograms/height in meters squared^16^; Blood pressure was registered according to the National Institute for Health and Clinical Excellence guidelines^48^.

### Statistical analyses

In the current report, we performed standard calculations of point prevalence for a representative sample, as the number of people in the sample with T2D and clinical depression comorbidity, or these diseases TA or nonadherence, divided by the total number of people in the population of SALUD Sector II (objective 1). Secondly, a bivariate analysis of the study primary outcomes and covariates with TA was carried out through chi-square test for categorical variables and Student’s t-test for continuous variables, with Levene’s test to test homogeneity of variance; in the case of a non-normal distribution, Mann–Whitney U and Kruskal–Wallis techniques were utilized (objective 2). Further analyses for risk estimation were first completed with bivariate binary logistic regression (LR) models. Finally, a hierarchical multivariate LR was performed stratifying models by hypothesis-guided covariate groups; in those models, all variables were included as continuous when possible to avoid data loss and/or the confounding effect due to variables categorization (objective 3). We included overweight/obesity (trough stratified IMC), daily smoking, and hypertension (high blood pressure) in the equation because they have recently been identified as shared and unique risk factors of both DM and depressive disorders, and we wanted to test their potential confounding effect in the association between primary outcomes (HbA1c and LDL-C) and nonadherence in patients with T2D and depression comorbidity. Diabetes and depression duration were selected for the same reason (potential confounding effect). Furthermore, we selected # drug intake, and the number of outpatient visits to nursing or primary care specialist (PCS) as covariates for two different hypothesis-guided reasons, firstly because they are health-state indicators and behaviours that may mediate the association between primary outcomes and treatment adherence, and secondly, we included these measures of health resources utilization as economic burden proxies. Statistical calculations were completed with IBM SPSS 22.0, licensed by the University of Zaragoza.

### Ethical concerns and considerations

The study design and procedures were previously evaluated and furtherly approved by SALUD Sector II Health Research Commission, the Secretary of the Quality of Care and Assistance Unit of SALUD and authorized by the Clinical Ethical Research Committee of Aragon, Spain (CEICA, Exp. CP-CI-PI17-0167) and was designed according to ethical standards as indicated in the Declaration of Helsinki and its subsequent amendments. Patient safety and data confidentiality has been fully guaranteed, maintaining at all times the anonymity of the patients, coding the cases without names or any identification that could link them to the population. This secondary study report population database was issued from SALUD public health services after the study design and procedures fulfilled formal and legal procedures and were approved by the above-mentioned Commissions and Ethical Committee. In this regard, informed consent was not required following recent bioethics recommendations since this report study design and research is solely based in secondary data, being clearly benign and has been proven to have no negative effects on clinical or other outcomes or values that matter to patients, proceeding without consent but with "public notification to the patient community in the healthcare services”^49,50^.

## Data Availability

The datasets generated during and/or analysed during the current study are available from the corresponding author on reasonable request.

## References

[1] International Diabetes Federation. *IDF Diabetes Atlas* (8th edition), https://www.idf.org/e-library/epidemiology-research/diabetes-atlas.html (International Diabetes Federation, 2017).

[2] Alberti KG, Zimmet PZ (1998). Definition, diagnosis and classification of diabetes mellitus and its complications. Part 1: diagnosis and classification of diabetes mellitus provisional report of a WHO consultation. Diabetes Med..

[3] Nathan DM (1993). Long-term complications of diabetes mellitus. N. Engl. J. Med..

[4] Nathan DM (2009). Translating the A1c assay into estimated average glucose values. Diabetes Care.

[5] Ho PM (2006). Effect of medication nonadherence on hospitalization and mortality among patients with diabetes mellitus. Arch. Intern. Med..

[6] Anderson RJ, Freedland KE, Clouse RE, Lustman PJ (2001). The prevalence of comorbid depression in adults with diabetes—a meta-analysis. Diabetes Care.

[7] Mezuk B, Eaton WW, Albrecht S, Golden SH (2008). Depression and type 2 diabetes over the lifespan: a meta-analysis. Diabetes Care.

[8] De Jonge P, Roy JF, Saz P, Marcos G, Lobo A (2006). Prevalent and incident depression in community-dwelling elderly persons with diabetes mellitus: results from the ZARADEMP project. Diabetologia.

[9] Hoogendoorn CJ, Roy JF, Gonzalez JS (2017). Shared dysregulation of homeostatic brain-body pathways in depression and type 2 diabetes. Curr. Diab. Rep..

[10] Campayo A (2010). Depressive disorder and incident diabetes mellitus: the effect of characteristics of depression. Am. J. Psychiatr..

[11] Renn BN, Feliciano L, Segal DL (2011). The bidirectional relationship of depression and diabetes: a systematic review. Clin. Psychol. Rev..

[12] Verma SK (2010). Impact of depression on health-related quality of life in patients with diabetes. Ann. Acad. Med. Singapore.

[13] Markowitz S, Gonzalez JS, Wilkinson JL, Safren SA (2011). Treating depression in diabetes: emerging findings. Psychosomatics.

[14] Pouwer F, Nefs G, Nouwen A (2013). Adverse effects of depression on glycemic control and health outcomes in people with diabetes: a review. Endocrinol. Metab. Clin. North Am..

[15] Hoogendoorn CJ (2019). Depressive symptom dimensions and medication non-adherence in suboptimally controlled type 2 diabetes. J. Diabetes Comp..

[16] World Health Organization. *Body Mass Index*. Available from: http://www.euro.who.int/en/health-topics/disease-prevention/nutrition/a-healthy-lifestyle/body-mass-index-bmi (2019). Accessed February 27 2021.

[17] Catapano AL (2011). ESC/EAS guidelines for the management of dyslipidaemias: the task force for the management of dyslipidaemias of the European Society of Cardiology (ESC) and the European Atherosclerosis Society (EAS). Atherosclerosis.

[18] Grundy SM (2013). An international atherosclerosis society position paper: global recommendations for the management of dyslipidemia. J. Clin. Lipidol..

[19] Roy JF (2010). Cardiovascular burden and long-term risk of first-ever depression: implications for the vascular depression hypothesis from a population-based study. J. Psychosom. Res..

[20] Lobo-Escolar A (2008). Association of hypertension with depression in community-dwelling elderly persons: results from the ZARADEMP project. Psychother. Psychosom..

[21] Grossman A, Grossman E (2017). Blood pressure control in type 2 diabetic patients. Cardiovasc. Diabetol..

[22] Chireh B, D’Arcy C (2019). Shared and unique risk factors for depression and diabetes mellitus in a longitudinal study, implications for prevention: an analysis of a longitudinal population sample aged⩾ 45 years. Ther. Adv. Endocrinol. Metab..

[23] Nouwen A (2019). Longitudinal associations between depression and diabetes complications: a systematic review and meta-analysis. Diabetes Med..

[24] De-la-Camara C (2010). Influence of gender on baseline characteristics of depression and future risk for incident stroke an elderly population: results from the ZARADEMP project. J. Psychosom. Res..

[25] Hutter N, Schnurr A, Baumeister H (2010). Healthcare costs in patients with diabetes mellitus and comorbid mental disorders—a systematic review. Diabetologia.

[26] Egede LE, Nietert PJ, Zheng D (2005). Depression and all-cause and coronary heart disease mortality among adults with and without diabetes. Diabetes Care.

[27] Haynes RB (1979). Determinants of Compliance: The Disease and the Mechanics of Treatment. Compliance in Health Care.

[28] Mann D, Ponieman D, Leventhal H, Halm E (2009). Predictors of adherence to diabetes medications: the role of disease and medication beliefs. J. Behav. Med..

[29] Asche C, LaFleur J, Conner C (2011). A review of diabetes treatment adherence and the association with clinical and economic outcomes. Clin. Ther..

[30] DiMatteo MR (2004). Variations in patients' adherence to medical recommendations: a quantitative review of 50 years of research. Med. Care.

[31] Lingam R, Scott J (2002). Treatment non-adherence in affective disorders. Acta Psychiatr. Scand..

[32] Dall TM (2019). The economic burden of elevated blood glucose levels in 2017: diagnosed and undiagnosed diabetes, gestational diabetes mellitus, and prediabetes. Diabetes Care.

[33] Safren SA (2014). A randomized controlled trial of cognitive behavioral therapy for adherence and depression (CBT-AD) in patients with uncontrolled type 2 diabetes. Diabetes Care.

[34] Pouwer F (2003). Rates and risks for comorbid depression in patients with type 2 diabetes mellitus: results from a community-based study. Diabetologia.

[35] Lee YJ (2014). Factors associated for mild cognitive impairment in older Korean adults with type 2 diabetes mellitus. Diabetes Metab J..

[36] Trento M (2012). A cross-sectional survey of depression, anxiety, and cognitive function in patients with type 2 diabetes. Acta Diabetol..

[37] Al-Hayek AA (2012). Association between diabetes self-care, medication adherence, anxiety, depression, and glycemic control in type 2 diabetes. Saudi Med. J..

[38] Gonzalez JS, Tanenbaum ML, Commissariat PV (2016). Psychosocial factors in medication adherence and diabetes self-management: Implications for research and practice. Am. Pshycol..

[39] Johnson JA (2012). Controlled trial of collaborative primary care team model for patients with diabetes and depression: rationale and design for a comprehensive evaluation. BMC Health Servi Res..

[40] Patel PJ (2017). Multimorbidity and polypharmacy in diabetic patients with NAFLD. Implications for disease severity and management. Medicine.

[41] Agámez Paternina, A.P., Hernández Riera, R., Cervera Estrada, L., & Rodríguez García, Y. Factores relacionados con la no adherencia al tratamiento antihipertensivo. *Revista Archivo Médico de Camagüey* **12**(5), http://www.redalyc.org/articulo.oa?id=211116122009 (2008).

[42] Roy JF (2008). Psychosomatic implications in the longitudinal study of late-life depression in the community: the ZARADEMP project. J. Psychosom. Res..

[43] Santabarbara J (2010). History of stroke, incident depressive disorder and competing risk of death. J. Psychosom. Res..

[44] Vink NM, Klungel OH, Stolk RP, Denig P (2009). Comparison of various measures for assessing medication refill adherence using prescription data. Pharmacoepidemiol. Drug Saf..

[45] Andrade SE, Kahler KH, Frech F, Chan KA (2006). Methods for evaluation of medication adherence and persistence using automated database. Pharmacoepidemiol. Drug Saf..

[46] O'Shea M, Teeling M, Bennett K (2013). The prevalence and ingredient cost of chronic comorbidity in the Irish elderly population with medication treated type 2 diabetes: a retrospective cross-sectional study using a national pharmacy claims database. BMC Health Serv. Res..

[47] American Diabetes Association (2015). Standards of medical care in diabetes. Diabetes Care.

[48] National Clinical Guideline Centre (UK). *Hypertension: The Clinical Management of Primary Hypertension in Adults: Update of Clinical Guidelines 18 and 34,* https://www.ncbi.nlm.nih.gov/books/NBK83274/ (London: Royal College of Physicians, 2011). 22855971

[49] Hamel MB, Faden RR, Beauchamp TL, Kass NE (2014). Informed consent, comparative effectiveness, and learning health care. New Engl. J. Med..

[50] Rebers S, Aaronson NK, van Leeuwen FE (2016). Exceptions to the rule of informed consent for research with an intervention. BMC Med. Ethics.

